# Comparative Genomics, Phylogenetics, Biogeography, and Effects of Climate Change on *Toddalia asiatica* (L.) Lam. (Rutaceae) from Africa and Asia

**DOI:** 10.3390/plants11020231

**Published:** 2022-01-17

**Authors:** Elizabeth Syowai Mutinda, Elijah Mbandi Mkala, Xiang Dong, Jia-Xin Yang, Emmanuel Nyongesa Waswa, Consolata Nanjala, Wyclif Ochieng Odago, Guang-Wan Hu, Qing-Feng Wang

**Affiliations:** 1 CAS Key Laboratory of Plant Germplasm Enhancement and Specialty Agriculture, Wuhan Botanical Garden, Chinese Academy of Sciences, Wuhan 430074, China; elizabeth@wbgcas.cn (E.S.M.); mkala@wbgcas.cn (E.M.M.); directx0831@163.com (X.D.); yangjxgz@163.com (J.-X.Y.); waswa.emmanuell@gmail.com (E.N.W.); Nanjala.conso@mails.ucas.ac.cn (C.N.); wyclifodago88@gmail.com (W.O.O.); qfwang@wbgcas.cn (Q.-F.W.); 2 Sino-Africa Joint Research Center, Chinese Academy of Sciences, Wuhan 430074, China; 3 University of Chinese Academy of Sciences, Beijing 100049, China; 4 East African Herbarium, National Museums of Kenya, Nairobi 451660-0100, Kenya

**Keywords:** Rutaceae, comparative analysis, phylogeny, biogeography, divergent hotspots

## Abstract

In the present study, two samples of *Toddalia asiatica* species, both collected from Kenya, were sequenced and comparison of their genome structures carried out with *T. asiatica* species from China, available in the NCBI database. The genome size of both species from Africa was 158, 508 base pairs, which was slightly larger, compared to the reference genome of *T. asiatica* from Asia (158, 434 bp). The number of genes was 113 for both species from Africa, consisting of 79 protein-coding genes, 30 transfer RNA (tRNA) genes, and 4 ribosomal RNA (rRNA) genes. *Toddalia asiatica* from Asia had 115 genes with 81 protein-coding genes, 30 transfer RNA (tRNA) genes, and 4 ribosomal RNA (rRNA) genes. Both species compared displayed high similarity in gene arrangement. The gene number, orientation, and order were highly conserved. The IR/SC boundary structures were the same in all chloroplast genomes. A comparison of pairwise sequences indicated that the three regions (*trnH-psbA*, *rpoB*, and *ycf1*) were more divergent and can be useful in developing effective genetic markers. Phylogenetic analyses of the complete cp genomes and 79 protein-coding genes indicated that the *Toddalia* species collected from Africa were sister to *T. asiatica* collected from Asia. Both species formed a sister clade to the Southwest Pacific and East Asian species of *Zanthoxylum*. These results supported the previous studies of merging the genus *Toddalia* with *Zanthoxylum* and taxonomic change of *Toddalia asiatica* to *Zanthoxylum asiaticum*, which should also apply for the African species of *Toddalia*. Biogeographic results demonstrated that the two samples of *Toddalia* species from Africa diverged from *T. asiatica* from Asia (3.422 Mya, 95% HPD). These results supported an Asian origin of *Toddalia* species and later dispersal to Africa and Madagascar. The maxent model analysis showed that Asia would have an expansion of favorable areas for *Toddalia* species in the future. In Africa, there will be contraction and expansion of the favorable areas for the species. The availability of these cp genomes will provide valuable genetic resources for further population genetics and biogeographic studies of these species. However, more *T. asiatica* species collected from a wide geographical range are required.

## 1. Introduction

The family Rutaceae contains flowering plants belonging to the order Sapindales. It comprises about 150–165 genera and approximately 2100 species widely distributed in the tropical and subtropical regions. The three main centers of its distributions are tropical America, southern Africa, and Australia [1,2,3,4,5,6]. *Zanthoxylum* is the second largest genus of Rutaceae, consisting of approximately 225 species, and it has a worldwide distribution [7,8].

Rutaceae has traditionally been divided into (two, three, four, or seven) subfamilies [3,6,9,10]. Amyridoideae is the largest and diverse subfamily based on the current circumscription [10]. In this subfamily, four genera *Phellodendron* Rupr., *Tetradium* Lour., *Toddalia* Juss., and *Zanthoxylum* L. were found to produce alkaloids [8,11,12] and the relationship of these chemical constituents has been hypothesized for the past years. The ancestor for Rutaceae has been linked to the several alkaloids recognized in these four genera, thus naming the group to date proto-Rutaceae [13]. *Toddalia* is a monotypic genus with one species *T. asiatica* (L.) Lam., widely distributed in Africa and Asia. 

The four groups have been widely studied in previous studies [3,14]. The results indicated that *Zanthoxylum* formed a sister group with *Toddalia*. However, it was evidenced that the genus *Toddalia* was nested within *Zanthoxylum* using the highest sampled taxa of *Zanthoxylum* [15]. Additionally, studies have proposed the change of *T. asiatica* to *Zanthoxylum asiaticum* (L.) Appelhans, Groppo & J. Wen, comb. Nov [8]. Moreover, the current research conducted using 36 complete plastomes supported *Toddalia* as part of *Zanthoxylum* and suggested that they should be merged [6]. Nevertheless, variations were observed. In a study carried out by Appelhans, *Toddalia* was a sister to the *Zanthoxylum* species found in Africa and Madagascar [8]. In another study, it was established that *Toddalia* was sister to the main Asian clade [16]. However, with the current study using 36 complete chloroplast genomes, *T. asiatica* was not closely related to the African and Malagasy species. However, it formed a sister clade with Southwest Pacific and the East Asian species of *Zanthoxylum* with a high support clade value.

**Figure 1:**
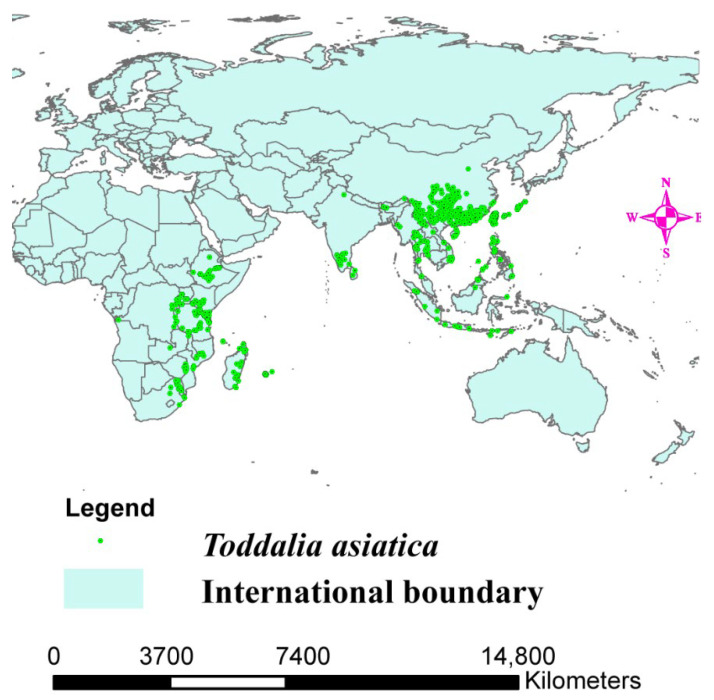
Distribution map of *Toddalia* asiatica in Africa and Asia.

The range of *Toddalia asiatica* may directly change because of altered climate, resulting in a change in its distribution (Figure 1). Projecting the favorable climatic niche for these species and investigating their control range is important for estimating the effects of climate change on the species as well as knowing the conservation measures to put in place. A previous study conducted indicated that vegetation growth will be affected by climate change resulting in the loss of germplasm resources and different types of species [17]. These continuous deviations in precipitation and climate will be the major basis for the extinction of most species in the world [18]. Additionally, migration and extinction of some organisms can be a result of climate volatility, affecting ecosystem patterns in the future [19,20,21]. Thus, future projections of several global trends using climate change models are necessary for evaluating the perception of how species are distributed and for the development of better conservation methodologies. The objectives of this study were to (1) sequence the two samples of *T. asiatica* species collected from Kenya, and compare their structural features with *T. asiatica* from Asia available in the NCBI database, (2) determine the phylogenetic placement of *T. asiatica* samples from Africa in the phylogenetic tree, (3) determine the origin of the genus *Toddalia* and its dispersal areas, and (4) determine the impacts of climate change on *Toddalia* species.

## 2. Results

### 2.1. Complete Chloroplast Genome

**Figure 2:**
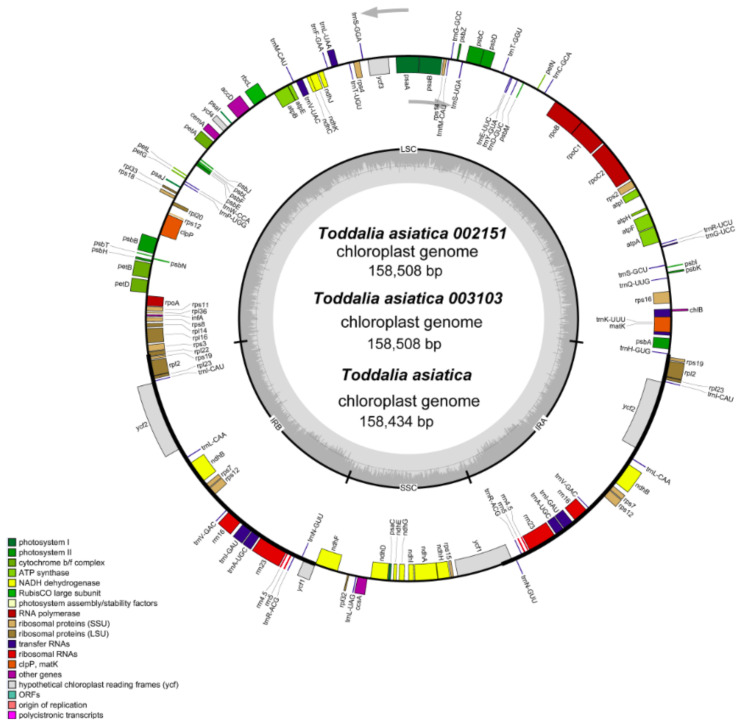
Gene map of *Toddalia* plastomes. Genes in the circle are transcribed clockwise, while the rest are transcribed counterclockwise. Dark gray shading in the inner circle indicates the GC content.

Using the advanced sequencing technology, the total DNA of the two samples of *Toddalia* species was isolated. The total pair of reads used in *T. asiatica* 002151 were 12,927,870 with an average embplant_pt kmer-coverage of 145.7 bp and average embplant_pt base-coverage of 475.1 while *T. asiatica* 003103 had a total of 817,3501 paired reads with an average embplant_pt kmer-coverage of 141.2 and average embplant_pt base-coverage of 460.6 depth over the chloroplast genomes. The number of annotated genes for these samples is shown (Table 1). They had a total of 113 genes including 79 protein-coding genes, 30 tRNA genes, and 4 rRNA genes. The total length of the cp genomes (*T. asiatica* 002151 and *T. asiatica* 003103), GC content, number of genes, and other information are shown (Table 2). The cp genome size for the sequenced samples was 158, 508 bp (Figure 2), which was slightly larger compared to the published genome of *T. asiatica* (158,434) collected from China. The cp genomes displayed a typical quadripartite structure consisting of a pair of IRs (27,007) separated by one LSC (86,162) and one SSC (18,332) region. The gene content for these samples was 38.5% indicating a close relationship of the species. The length of exons and introns for the species and splitting genes are indicated (Supplementary Table S1). Introns are essential features that take part in the regulation and signaling of the expression of genes within the species [22]. Carrying out a comparative analysis of the chloroplast cp genomes is important as it relays useful information on the present functional genes, genetic mutations, and rearrangements that are key drivers of evolution [23].

The DNA gene content (GC) is an important factor that implies the closeness of species. The GC content of *Toddalia* species compared was 38.5%, which was lower than the AT content. The average GC contents of the LSC region, IR region, and SSC regions were 36.8%, 42.9%, and 33.4%. This indicates that the rRNA and tRNA, which occupy mainly IR regions, prefer to use bases G and C. However, the IR regions depicted a higher GC content of 42.9% and this is attributed to the presence of the rRNA and tRNA genes within the inverted repeat region, which occupies a greater area than the protein-coding genes. This occurrence has also been displayed in other studies [24]. 

### 2.2. Codon Preference Analysis

Codon usage is an important aspect of gene expression in the species genomes [25]. Different species tend to use some codons often while other codons are rarely used. This indicates that different organisms vary in their synonymous codon rates of occurrences in their protein-coding sequences. Genes in the closely related species generally show a similar codon use pattern. Through the analysis of codon preference, we can better understand the evolution of species [26]. Based on the protein-coding sequences, the frequency of codon usage was estimated for *Toddalia* plastomes. In total, the protein-coding genes were composed of 52,811 for *T. asiatica* and 52,836 for both *T. asiatica* 002151 and *T. asiatica* 003103, which encoded 21 amino acids (Supplementary Table S2). Among them, the most frequent amino acid was leucine 10.24% for *T. asiatica* 002151, 10.20% for *T. asiatica* 003103, and 10.01% for *T. asiatica*. The least frequent coded amino acid was cysteine with 1.385% for *T. asiatica* 002151, 1.313% for *T. asiatica* 003101, and 1.351% for *T. asiatica*. In our analysis, the relative synonymous codon usage (RSCU) values of 35 codons were greater than 1 and most of them ended with A or U and only five codons ended with G. This indicated that the preferred codons tended to be A/U ending. This phenomenon was the same as that reported for other Rutaceae plastomes [27]. The choice of codon usage is a result of mutation and selection factors [28]. Thus, the choice of codons within the plastome can be used to show gene expressions and speciation mechanisms in species.

### 2.3. IR Contraction and Expansion

**Figure 3:**
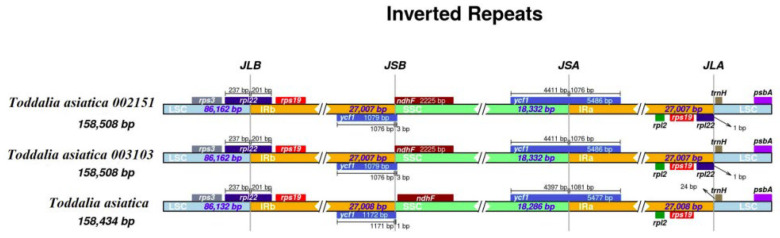
Comparison of the borders of large single copy (LSC), small single copy, (SSC), and inverted repeat (IR) regions among the *Toddalia* plastomes. Different color boxes indicate specific genes.

The contraction and expansion of the IR region at the borders affect the size difference between chloroplast genomes and play important roles in evolution [29]. Although the IR regions of these species were relatively conserved, some structural variations were noted in *T. asiatica* 002151 and *T. asiatica* 003103 from Africa compared to the reference species from Asia (Figure 3). The IR regions for the plastomes were 27,008 bp in length for *T. asiatica* and 27,007 bp in length for *T. asiatica* 002151 and *T. asiatica* 003103. In these species, genes *rpl*22 and *ycf*1 extended in the LSC/IRb and SSC/IRa borders. Gene’s *rps*3, *rps*19, *rpl*2, *rpl*22, *trnH*, and *psb*A were found in the LSC/IRb and IRa/LSC region while *ndh*F was found in the IRb/SSC region. In the two samples of *Toddalia* species from Africa, the gene *rpl22* was found in the LSC/IRb and IRa/LSC regions. All the plastomes had similar SSC/IRa borders and the junction was all crossed by the gene ycf1 with a length from 1076 to 1081 bp. In addition, it was found that the gene *rpl2* was found in all the species. Inverted repeats are the most conserved regions of the chloroplast genome [30] and great variations occur in land plants [31]. They play a vital role in the stabilization of the plastome [32] and also affect their size [33].

### 2.4. Repeat Analysis

**Figure 4:**
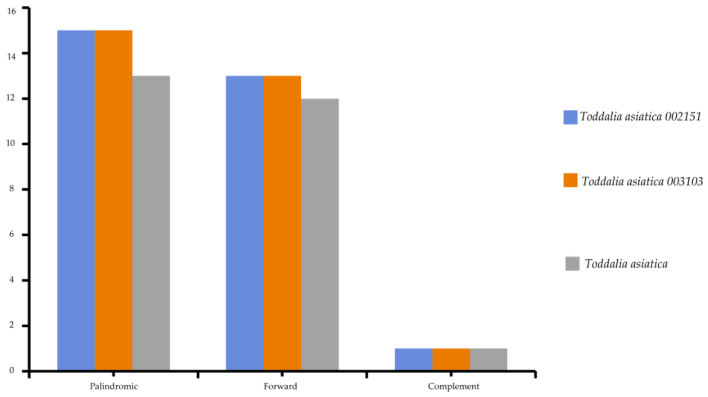
Total number of repeats found in *Toddalia* species.

Chloroplast repeats are major genetic resources that take a vital role in the rearrangement and recombination of the genomes [34]. They are useful for carrying out biogeographical and population genetic studies [35]. The plastomes of the species used in this study were found to contain a varied number of repeats (i.e., palindromic, forward, and reverse repeats). 81 repeats was recorded comprising of 43 palindromic repeats, 38 forward repeats, and 3 complementary repeats (Figure 4). The long repeats in these *Toddalia* species ranged from 30 to 73 bp in length. The long repeat length of 30 bp was dominant and occurred in all the species’ cp genomes (Supplementary Table S3). The number of tandem repeats was 25 in *T. asiatica*, comprising of 13 palindromic repeats, 12 forward repeats, and a complement repeat. A total of 28 repeats in both *T. asiatica* 002151 and *T. asiatica* 003103 consisting of 15 palindromic repeats, 13 forward repeats, and a complement repeat were recorded.

**Figure 5:**
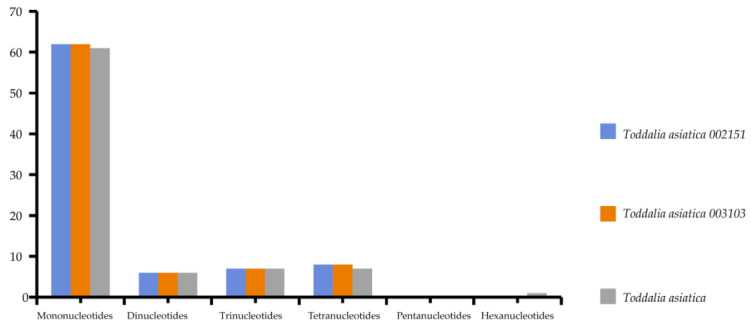
The total number of simple sequence repeats present in *Toddalia* species.

Simple sequence repeats (SSRs) are short repeat motifs of DNA sequences that normally show high levels of polymorphism, even between closely related species. Chloroplast SSRs are potentially useful molecular markers for population genetics and polymorphism studies [36,37,38,39,40,41]. There were no great variations found in the number of SSRs calculated in these species. Mononucleotides to tetranucleotides were present in all the samples. Pentanucleotides were missing in all the species while there was only one hexanucleotide in *T. asiatica*. Among these SSRs, mononucleotides reported the highest repeat motifs (Supplementary Table S4). *Toddalia asiatica* from Asia had 82 SSRs comprising of 61 mononucleotides, 6 dinucleotides, 7 trinucleotides and tetranucleotides, and 1 hexanucleotide. Both *T. asiatica* 002151 and *T. asiatica* 003103 from Africa had 83 SSRs with 62 mononucleotides, 6 dinucleotides, 7 trinucleotides, and 8 tetranucleotides (Figure 5, Supplementary Table S5). Although the genes within the chloroplast plastomes are highly conserved, the number of SSRs differs among and within the species [41]. Among these samples of cp plastomes, A/T mononucleotide repeats were the highest and these findings were in concordance with other studies [42,43]. However, other studies have indicated di-nucleotides and tri-nucleotides as the highest [35,44]. Our findings demonstrated that SSRs within these chloroplast plastomes majorly consist of A/T repeats. This is attributed to the A/T abundance of the species cp plastomes. This phenomenon has been observed in many previous studies [45,46]. Therefore, SSRs are important for understanding intrageneric and intergeneric differences within species.

### 2.5. Comparative Analysis

**Figure 6:**
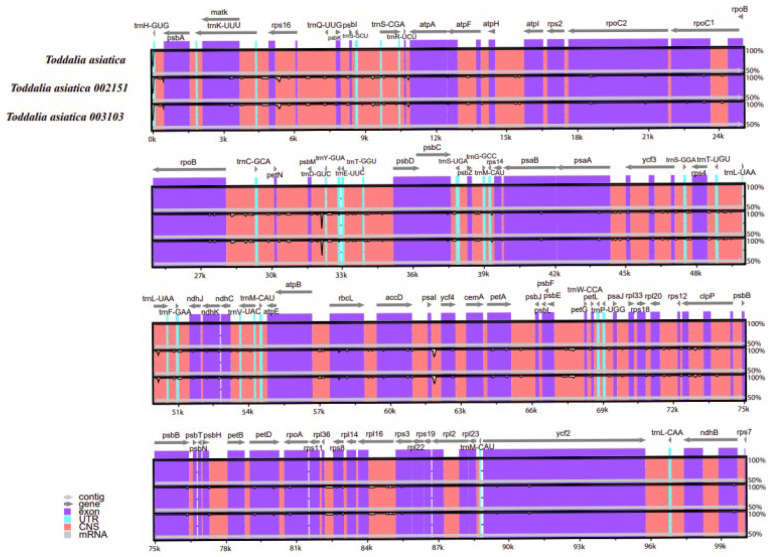
Sequence alignment of plastomes of *Toddalia* species using the LAGAN method. A cut-off of 70% similarity was used for the plot and the y-scale represents the percent similarity ranging from 50–100%.

Comparison of the *Toddalia* samples was conducted using Mvista and with the annotation of *T. asiatica* as a reference. The comparison demonstrated that the plastomes had a high consistency in gene arrangement (Figure 6, Supplementary Figure S1). The results depicted that the gene number, orientation, and order were highly conserved. Most sequence variations occurred in the non-coding sequences, which indicated that the coding regions were more conserved than non-coding regions. The IR regions were much less divergent than the LSC and SSC regions. Similarly, non-coding regions had higher gene differences compared to the coding regions. Moreover, within the SSC and the LSC regions, the non-coding regions were more varied than the coding regions. This pattern has been observed in other studies [47]. The IR regions were more conserved based on the abundance and gene order. The differences in the size of these cp genomes could be a result of the contraction and expansion of the IR regions. DNA barcodes generally have a high mutation rate and can be utilized in the identification of species in a given taxonomic group [48,49,50] and they can be vital markers for evolutionary studies as well as barcoding.

### 2.6. Sequence Divergence Analysis

**Figure 7:**
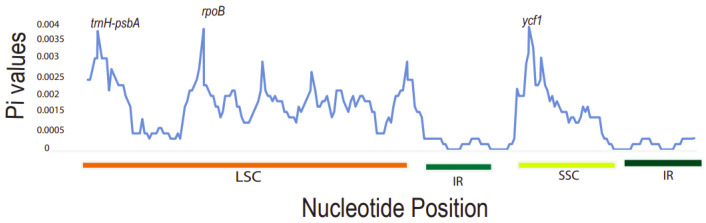
A sliding window analysis of nucleotide variability (Pi) values of different regions of *Toddalia*. *X*-axis: position of the midpoint of a window, *Y*-axis: nucleotide diversity of each window.

It was found that the Pi values ranged from 0 to 0.004. The three most divergent hotspot regions observed were *trnH-psb*A, *rpo*B, and *ycf*1 genes. They exhibited significantly higher Pi values (>0.0035) and were all located in the LSC and SSC region. Inverted repeat regions displayed lower nucleotide diversity, depicting that they are more conserved compared to the LSC and SSC regions (Figure 7). The large single-copy region (LSC) had the highest number of diverse regions followed by the SSC region. The dN/dS value can be used to measure the evolution rate of a specific gene [51]. This is the ratio of the synonymous substitution rate (dS) to the non-synonymous substitution rate (dN), which is important in analyzing the evolutionary pressures within the plastomes. Synonymous substitutions are more frequent than non-synonymous ones in protein-coding sequences [52]. These varied regions may be undergoing rapid nucleotide substitution at the species level. Thus, this demonstrates their ability as potential barcode markers for phylogenetic analysis studies and plant identification.

### 2.7. Phylogenetic Analysis

**Figure 8:**
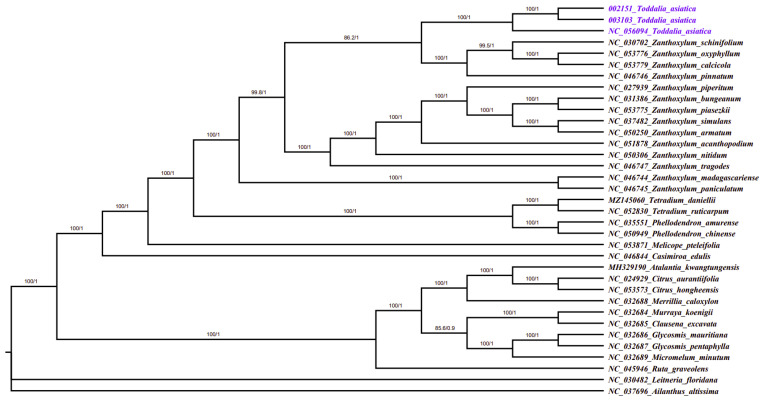
Phylogenetic tree construction of 35 taxa using maximum likelihood (ML) and Bayesian inference (BI) methods using 79 protein-coding genes.

Plastomes are vital features for comprehending intra-and interspecific evolutionary histories, and recent studies have shown their potential in phylogenetic, evolution, and molecular systematic analysis [30]. The more variable the sites in chloroplast plastomes are, the more potential they have in solving phylogenetic relationships within and among species [53,54]. Phylogenetic study of the two data sets using 35 complete plastomes and 79 protein-coding genes formed a well-resolved phylogenetic tree. The maximum likelihood (ML) phylogenetic tree using complete plastomes was congruent with the Bayesian inference (BI) tree using protein-coding genes with a high support value in almost every branch (Figure 8). The two samples (*T. asiatica* 002152 and *T. asiatica* 003103) collected from Kenya, Africa were a sister to *T. asiatica* collected from China, Asia. These *Toddalia* species formed a sister clade to *Z. calcicola*, *Z. oxyphyllum*, *Z. pinnatum*, and *Z. schinifolium* from the Southwest Pacific and East Asian species of *Zanthoxylum* with a high support value. These results were in agreement with previous studies [6]. The *Toddalia* species analyzed in this study clustered together in the same clade and formed a sister clade with *Zanthoxylum* species, thus showing a closer relationship of these species. Our results support previous studies [6,8] of merging the genus *Toddalia* with *Zanthoxylum*. However, more cp plastomes of the *T. asiatica* species are needed to understand the precise position of these species in the phylogeny. Inconsistencies in the topology of the tree may be attributed to low taxon sampling [55].

### 2.8. Divergence Time Estimation

**Figure 9:**
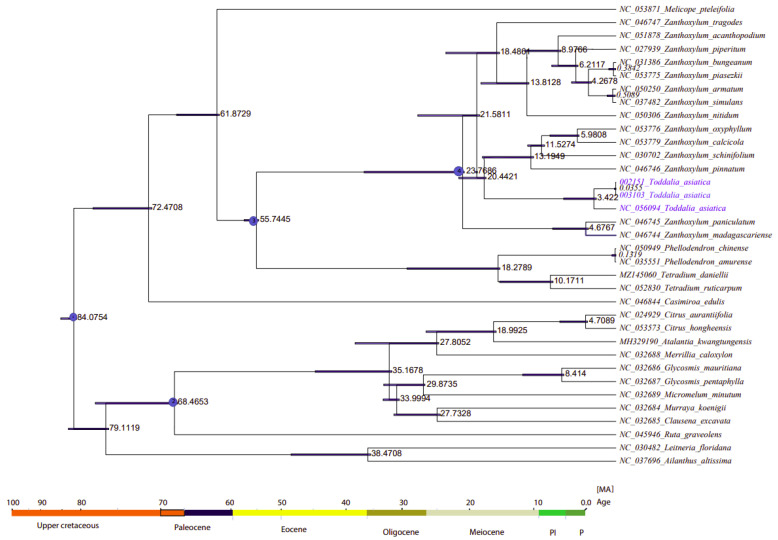
Phylogenetic chronogram showing the evolutionary dating time taxa of the 15 genera in the family Rutaceae and two outgroups. The tree was estimated using Bayesian analysis of 79 protein-coding genes and 35 taxa in the Markov Chain Monte Carlo (MCMC) tree.

The divergence time of Rutaceae was estimated at 85 million years ago (Mya) (HPD%) [56] (Figure 9, node 1). The two samples of *Toddalia* species (*T. asiatica* 002151 and *T. asiatica* 003103) from Africa diverged from *T. asiatica* from Asia 3.422 million years ago. These *Toddalia* species diverged from the Southwest Pacific and East Asian species of *Zanthoxylum* (*Z. pinnatum*, (*Z. schinifolium*, *Z. oxyphyllum*, and *Z. calcicola*)) at 20.4421 million years ago. Compared to the crown ages of the sister clade to *Toddalia*, *Toddalia* species are relatively recent and inadequate sampling of these species might have caused this difference. These *Toddalia* species and Southwest Pacific and East Asian species of *Zanthoxylum* formed a clade that diverged from the rest of *Zanthoxylum* at 21.5811 million years ago while *Zanthoxylum* species from Madagascar (*Z. paniculatum* and *Z. madagascariense*) diverged from the rest of *Zanthoxylum* at 23.7608 million years ago. The genera *Zanthoxylum*, *Toddalia*, *Phellodendron*, and *Tetradium* are known as the oldest fossils of the family Rutaceae [3,8]. The clade consisting of the genera *Phellodendron* and *Tetradium* diverged from *Zanthoxylum* at 55.7445 million years ago. On the other hand, *Phellodendron* diverged from *Tetradium* at 18.2789 million years ago. Although these results shed light on the biogeography of *T. asiatica* species, more of these species collected from a wide geographical area are needed in future studies.

### 2.9. Diversification Rates Analyses

**Figure 10:**
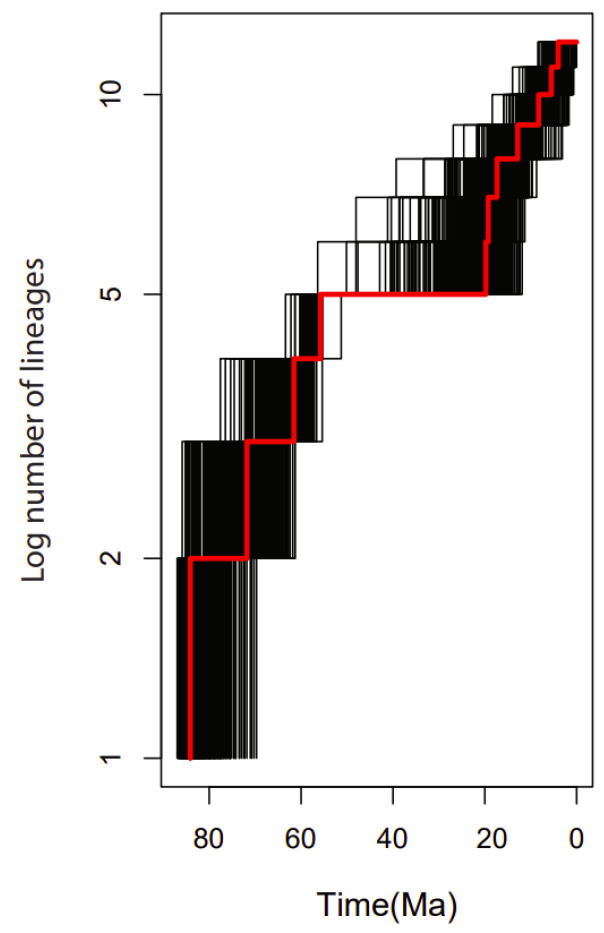
Lineage-through-time (LTT) plots and the rate shifts in the 15 genera used in the family Rutaceae. Black lines represent the LTT plots for 1000 trees randomly selected from the BEAST analysis. The red line shows the plot from the maximum clade credibility tree.

The lineage through time (LTT) plot showed an acceleration of the diversification rate of lineages within the 15 genera of the family Rutaceae mainly after 20 Mya (Figure 10).

### 2.10. Ancestral Area Reconstruction 

**Figure 11:**
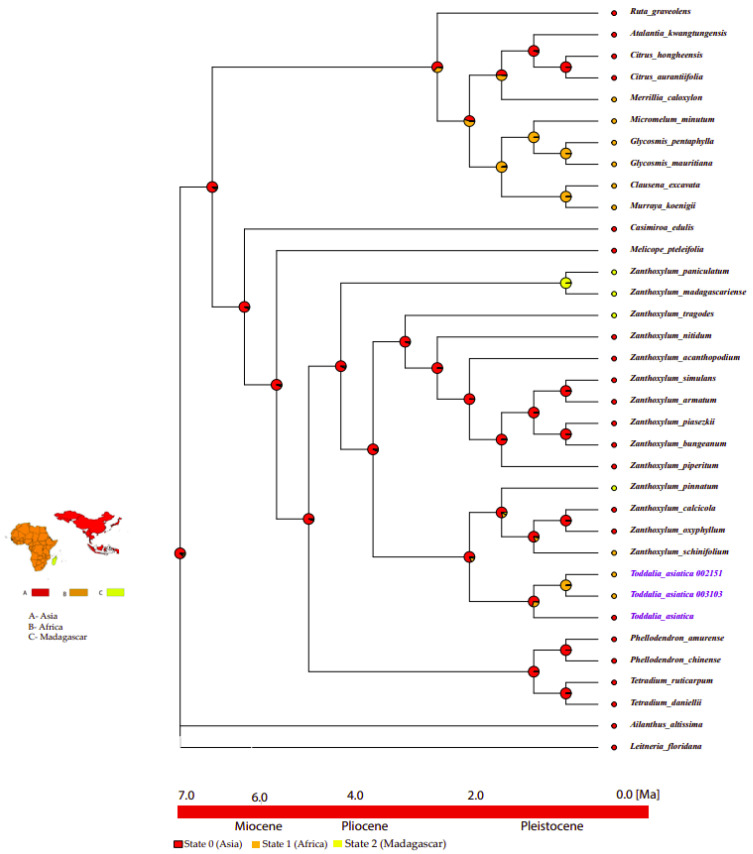
Ancestral area reconstructions of *Toddalia* species and closely related species based on the MBASR using R-software. The four biogeographical areas were defined based on the distribution of extant *T. asiatica* species. Area abbreviations: A, Asia; B, Africa; C, Madagascar. The base map was downloaded from Worldclim (https://www.worldclim.org, accessed on 26 September 2021).

Based on the topology of the interspecific chronogram of the MBASR analysis, it supported the ancestral distribution of *T. asiatica* species in the Asian continent (Figure 11, node 1). According to the data, we infer that *T. asiatica* species colonized the continent of Asia, and later dispersed to Africa and Madagascar. 

### 2.11. Climate Variables

**Figure 12:**
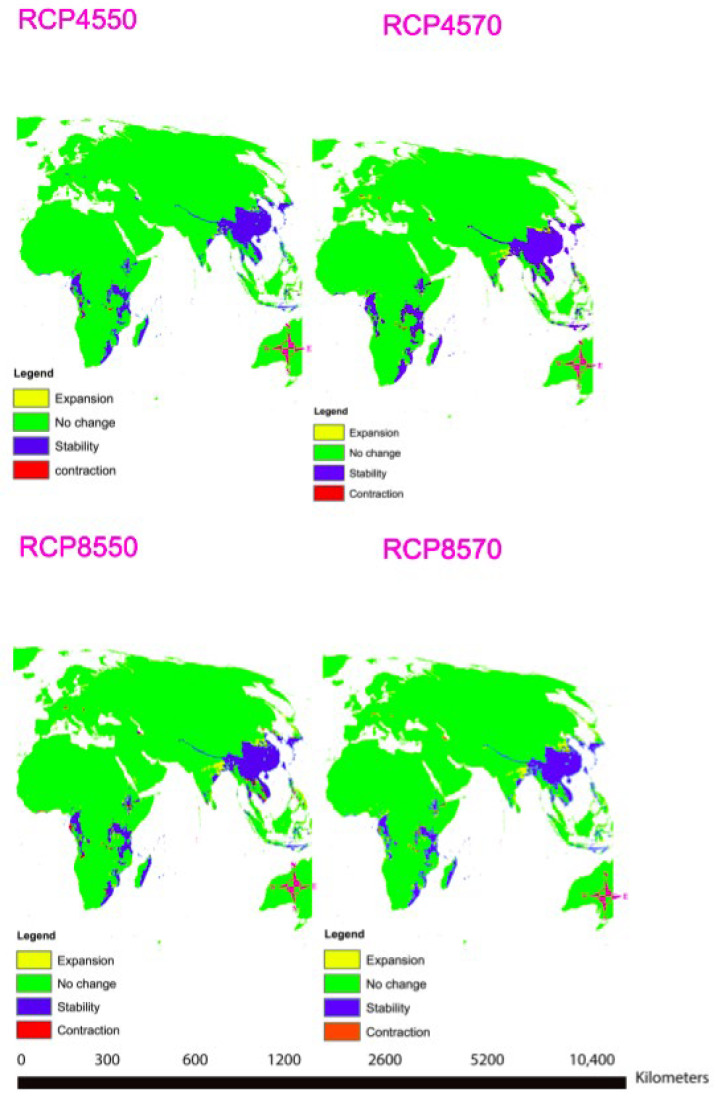
Maps showing distribution modeling for *Toddalia asiatica* for the years 2050 and 2070.

A total of nine important bio-climatic variables were obtained from the correlation of the 19 commonly used bio-climatic variables (Table 3). The most important factors for predicting the distribution of *T. asiatica* are Precipitation of the Wettest Month (bio13) and Mean Temperature of Driest Quarter (bio9) (Table 3, Supplementary Figure S2). The area under the curve (AUC) values were high, with an average mean of 0.98, which confirmed that the results obtained were excellent (Supplementary Table S6, Supplementary Figure S3). The projected future habitat of the *T. asiatica* indicates that the species is likely to shift due to climatic variable changes. Under climate projection in RCP 4.5 for the years 2050 and 2070, no much change will be observed for *Toddalia* species in Africa, but a slight expansion of the favorable areas will be observed in Asia. In RCP 8.5 for the year 2050 and 2070, projected as the extreme scenario (Figure 12), there will be more expansion of the favorable areas of these species in Asia in the future. However, these species will be lost in some parts of Africa due to the contraction of the favorable areas while in some parts there will be an expansion of the favorable areas. It is worth mentioning that during the pliocene, some relict niches were previously reported to occur in Europe, where *Toddalia* persevered under hostile factors until the late Pliocene, which resulted in the shift of this genus eastward and southward to Asia and Africa. This rapid shift and disappearance of *Toddalia* as a result of climate change in the early pliocene were subjected to humid conditions in Europe, which became colder in the late pliocene [57]. This is supported by our study as most of the species have been found to exist in Asia and Africa, where climatic conditions seem to favor the distribution of this genus.

## 3. Materials and Methods

### 3.1. Plant Materials Collection, DNA Extraction, and Sequencing

The fresh samples of *T. asiatica* sequenced in this study were collected from Mt. Kenya, Kenya (SAJIT-002151 *Toddalia asiatica* (L.) Lam. (Lat 0.1743; Lon 37.141; 2412 m)) and (SAJIT-003103 *Toddalia asiatica* (L.) Lam. (Lat 0.1065; Lon 37.145; 2392 m)) (Supplementary Table S7). They were preserved, using silica gel to dry. The voucher specimens were later stored at Wuhan Botanical Garden Herbarium (Wuhan, China). The DNA was isolated using the improved Cetyltrimenthylammonium bromide (CTAB) method [58]. The NanoDrop spectrometer (Beckman Coulter, Krefeld, Germany) and gel electrophoresis (Beijing Liuyi Instrument Factory, Beijing, China) were used in determining the quantity and quality of the isolated DNA material. The other 33 species in order Sapindales were downloaded from NCB1 (https://www.ncbi.nlm.nih.gov/, accessed on 6 August 2021, Supplementary Table S8).

### 3.2. Plastome Annotation and Assembly

The Cp genome was sequenced using the Illumina platform at Novo gene Company (Beijing, China).The low quality data and adaptors were later filtered, and the obtained clean data was assembled using GetOrganelle-1.7.2 software [32,59,60,61]. The mean and maximum size of the two samples of *Toddalia* were set at 150-bp for K-mer reads at K 21, 45, 65, 85, and 105. The bandage was used in visualizing the assembled graphs to validate the produced chloroplast genomes [62]. The trimmed raw reads were later aligned to the de novo assemblies based on the reading Geneious prime 2021 [63]. The low-sensitivity to medium option and iteration of five times were used [64]. CpGAVAS2 (http://www.herbalgenomics.org/cpgavas, accessed on 8 August 2021) was used in the annotation of sequences using the published genome of *T. asiatica* as a reference (NC_056094.1), which were later edited manually. The chloroplast genome CDs sequences were matched on NCBI and corrected using the online blast software version 2.2.25 (http://www.ncbi.nlm.nih.gov, accessed on 8 August 2021). The annotation of tRNA was performed using tRNAscan-SE software (http://trna.ucsc.edu/tRNAscan-SE/, accessed on 9 August 2021) [65]. The online program, OGDRAW (https://chlorobox.mpimp-golm.mpg.de/OGDraw.Html, accessed on 9 August 2021) [66], was applied in mapping the complete chloroplast genomes. The complete chloroplast genomes were deposited at the GenBank accession numbers (OK127881) for *T. asiatica* 002151 and (OK127881) for *T. asiatica* 003103. 

### 3.3. Repeat Analysis

REPuter (https://bibiserv.cebitec.uni-bielefeld.de/reputer, accessed on 16 August 2021), an online tool, was employed in the identification of long repeats (forward, reverse, palindromic, and complementary) with settings of 30 bp minimum repeat size, a hamming distance of 3, and sequence identity of not less than 90% [67]. A tandem repeat finder program (https://tandem.bu.edu/trf/trf.html, accessed on 16 August 2021) [68] was used in the identification of tandem repeats using parameters of two and seven for the matching alignment, mismatch, and Indels in that order. MISA (https://webblast.ipk-gatersleben.de/misa, accessed on 16 August 2021) [69], was also applied in the identification of simple sequence repeats (SSRs) with a threshold of 10 repeats for mononucleotides, five repeats for dinucleotides, four repeats for trinucleotides, and three repeats for both tetranucleotides, pentanucleotides, and hexanucleotides.

### 3.4. Comparative Analysis

The structural similarities and differences of the *Toddalia* species were compared using the mVISTA (https://genome.lbl.gov/vista/mvista/submit.shtml, accessed on 18 August 2021), an online tool with the Shuffle LAGAN model [70], and using the annotation of *T. asiatica* from Asia as the reference. The comparison between the boundaries of SSC, LSC, and the IR regions of these complete cp genomes was performed using chloroplot (https://irscope.shinyapps.io/chloroplot/, accessed on 18 August 2021), an online tool. The IR contraction and expansion of the IR/SC boundary regions in the cp genomes were visualized using IRscope (https://irscope.shinyapps.io/irapp, accessed on 18 August 2021) [71].

### 3.5. Selective Pressure Analysis

The nucleotide diversity (Pi) in the two samples of *Toddalia* species was investigated in a sliding window using DnaSP (http://www.ub.edu/dnasp, accessed on 18 August 2021) [72] and Microsoft Excel 2011 (Redmond, WA, USA) was used to convert the results into charts for observation.

### 3.6. Phylogenetic Analysis

A total of 35 complete plastomes from 15 genera of Rutaceae (two plastomes newly generated and 33 plastomes obtained from GenBank) were included for Phylogenetic analyses. Two species of Simaroubaceae (plastomes obtained from GenBank) were used as outgroups. The alignment of these sequences was performed in MAFFT [73], then the non-homologous sites were deleted for phylogenetic trees. Bayesian inference (BI) was executed with MrBayes v3.2.3 [74] in the GTR+G+I model. Maximum likelihood trees (ML) were carried out using IQtree [75] and 1000 bootstrap replicates were tested to evaluate the branch support values. The 79 protein-coding genes of the 35 sequences were also extracted for further phylogenetic tree analysis, employing similar methods to the complete chloroplast genome sequences. The CDs were then combined in a single file and aligned using MAFFT [73]. Geneious prime version 2021 was used to concatenate the sequence into various readable formats for other analyses. 

### 3.7. Divergence Time Estimation

The age estimates for the 15 genera in the family Rutaceae were executed in BEAST version 1.8.4 [76]. The concatenation matrix of the 79 CDs of 35 taxa was used to estimate the divergence time of *T. asiatica* and other different lineages in the family Rutaceae. The chronogram and branch intervals were linked to partitions and other constraints were unlinked. We used the Yule process prior for speciation and the uncorrelated lognormal relaxed clock model. Prior’s constraints time of the nodes were selected using the Log-Normal distribution of the mean and standard deviation set at the mean and median limits, and the GTR+I+G substitution model was set as the nucleotide substitution model. The calibration points for the tree chronogram of Rutaceae used were four in total. To estimate the most common recent ancestor of *Toddalia*, *Phellodendron*, and *Tetradium* and their age divergence, (Log-Normal priors, offset 48.0, M 0.5, S 1.0 and offset of 55.4, M 0.5, S 2) were used respectively [8]. The stem-group for all Rutaceae ingroups was assigned with a lognormal distribution, a mean of 0.5, a standard deviation of 2 MA, and an offset of 69.59 MA [56]. To evaluate the divergence time of the 15 genera used in family Rutaceae, the 95% highest posterior density (HPD) limit of crown age was estimated at 84 Mean (M), Sigma (S) 1.0. To estimate the dating time, analyses were performed using BEAST (old) on XSEDE version 1.8.4 on the CIPRES web server (https://www.phylo.org/portal2/home.action, accessed on 26 September 2021). The MCMC analyses were run for 150 million generations, with sampling every 1000 generations. To check for repeatability, uniformity, and the coalescent model, parameters in each run were performed using BEAST. To check the burn-in of trees and chains distribution, we used tracer version 1.7.1. Tree Annotator version 1.8.0 was used to obtain a readable file for the tree. To show the mean divergence time approximates, 95% HPD intervals was used. Fig tree version1.4.4 was used to read the obtained file and visualize the tree.

### 3.8. Diversification Rates Analyses 

The ape package [77] in R version 4.0.3 [78] was used to calculate the lineage-through-time (LTT) plots to deduce the rates of diversification within the 15 genera in family Rutaceae.

### 3.9. Ancestral Area Reconstruction of Toddalia 

MBASR (an acronym for the MrBayes Ancestral States with R) was used to evaluate the likely ancestral areas of *T. asiatica* with the trees attained from the BEAST analysis [79]. We converted the phylogenetic tree in Figtree to Newick format. We then saved the tree in the input files as mb and saved it as mb.exe for windows. Three biogeographic areas were elucidated according to the distribution of existing *T. asiatica* species: A, Asia; B, Africa; C, Madagascar. To determine the precise distribution areas of the species, occurrences of species of *T. asiatica* from the specimen information were filtered and re-identified. Data was obtained from Global Biodiversity Information Facility (GBIF, http://www.gbif.org/, accessed on 12 September 2021). We prepared traits data files using identical names of the 35 taxa and assigned them scores as 0, 1, and 2 representing different states of the species collected, and missing scores were coded in a question mark (?). The results were written as a tree plot. 

## 4. Species Distribution Data

The Global Biodiversity Information Facility (GBIF, https://www.gbif.org, accessed on 12 September 2021) was used to obtain data on the occurrence of *T. asiatica* (Figure 1). A total of 3493 distribution points of *T. asiatica* in Africa, Asia, and Madagascar were obtained. To ensure the sample data was accurate, the designated sampling points were filtered, and arc map 10.8 was applied to plot the data. To ascertain the mapped points, we used the command function in R software to plot the same data. The data were filtered using the spin function command, and the occurrence data was rarefied at a distance of 30 km to reduce biases. The occurrence data was reduced to 1794 distribution points of the *T. asiatica* species from 3493 distribution points.

### 4.1. Environmental Factors Consideration

A total of 19 important environmental variables were used in modeling the projections of species distributions [80]. Some packages provide the most applied bioclimatic variables, which contribute to the development of the interpolation routines for the preparation of frequently used sources of bioclim data (Worldclim) [3,81]. The highest-resolution data (10 min per arc (approximately 17 km)) of the 19 bioclimatic variables were obtained from the Worldclim database. To ensure the sample data is accurate, the designated sampling points were filtered, and arc map 10.8 was applied to plot the data. The Worldclim dataset used R software (R Foundation, Vienna, Austria) command line using raster, usdm, dismo packages, and others were used in the analysis. The collinearity of the variables was analyzed using the usdm package and vifstep with a threshold of 0.7 to reduce the occurrence of over-fitting and to increase the testing accuracy. Nine bioclimatic variables were obtained from the correlation analyses of the 19 bioclimatic considerations and employed for further analyses (Table 3).

### 4.2. MaxEnt Model

MaxEnt algorithm version 3.3.3 [82], was applied in running all the models used in the study with default settings. The probability maps for habitat suitability and 10 replicates were used [83]. The MaxEnt model is best used when the data points consist of presence-only and with few numbers of records [83,84,85]. The training and test data points used were 70% and 30%, in that order. The relative importance of each environmental predictor for the models of *T. asiatica* was obtained using the percent contribution of the Jackknife test [82], which is the suitable index for small sample sizes [86]. The area under the curve (AUC) of the receiver operating characteristic curve (ROC) was calculated to find out the accuracy of the resulting models. The AUC score, a dominant tool, is favorable for measuring the model performance, because of its independence of threshold choices [85,87,88]. The performance of the model is best when the AUC value is higher or closer to 1 (Fielding and Bell, 1997; Phillips et al., 2006). The produced AUC graph is attained by plotting the true positive predictions (sensitivity) against the false-positive predictions (1-specificity) [89]. Additionally, the minimum difference between training and testing AUC data was also considered; a smaller variation signifies less overfitting present in the model [85,90]. The logistic output of the MaxEnt application is a map, indexing the environmental suitability of *T. asiatica*.

## 5. Conclusions

In this study, using next-generation sequencing technology, we successfully sequenced the whole chloroplast genome for the samples of *T. asiatica* collected from Kenya, Africa. In comparing these *Toddalia* species with the reference genome (*T. asiatica*) in the NCBI database, they showed similarities in terms of their composition and genomic structure. Both *Toddalia* species were represented with strongly supported phylogenetic trees using ML and BI analysis. The results supported the merging of the genus *Toddalia* with *Zanthoxylum* and taxonomic change of *Toddalia asiatica* to *Zanthoxylum asiaticum*. In addition, they provided useful genetic information for future studies of their chloroplast sequence variations, assembly, and evolution. Large repeats, SSRs, and variable loci are important genetic resources that can be employed as markers for barcoding. Biogeographic analysis indicated an Asian origin of *T. asiatica* species. MaxEnt models showed that Asia would have a favorable climate for these species in the future. In Africa, there will be contraction and expansion of the favorable areas of the species. Assessing the role of climate change in shaping the spatiotemporal patterns of these species in terms of their diversity, genetic diversity, and phylogenetic diversity will be ideal research in future studies. Climate change studies are also important for knowing the conservation measures of species to prevent their extinction. More studies are also required for the genome structures and phylogeography of *Toddalia* species by adequate sampling within their distribution range.

## Figures and Tables

**Table 1:** List of annotated genes of *T. asiatica*.

Gene Group	Gene Name
Ribosomal RNAs	*rrn1*6(2), *rrn*23(2), *rrn*5(2), *rrn*4.5(2)
Transfer RNAs	trnA-UGC*(2), *trnC-GCA*, *trnD-GUC*, *trnE-UUC*, *trnF-GAA*, *trnG-GCC*, *trnH-GUG*, *trnS-CGA*, trnK-UUU*, *trnL-CAA*(2), trnL-UAA*, *trnL-UAG*, *trnM-CAU*, *trnN-GUU*(2), *trnP-UGG*, *trnQ-UUG*, *trnR-ACG*(2), *trnR-UCU*, *trnS-GCU*, *trnS-GGA*, *trnS-UGA*, *trnT-GGU*, *trnT-UGU*, *trnV-GAC*(2), trnV-GUA*, *trnW-CCA*, *trnY-GUA*
Proteins of small ribosomal subunit	*rps*16*, *rps*2, *rps*14, *rps*15, *rps*4, *rps*7(2), *rps*18, *rps*12* (2), *rps*11, *rps*8, *rps*3, *rps*19(2)
Proteins of large ribosomal subunit	*rpl*33, *rpl*20, *rpl*36, *rpl*14, *rpl*16*, *rpl*22, *rp*l2* (2), *rpl*23(2), *rpl*32
Subunits of RNA polymerase	*rpoC*2, *rpoC*1*
Photosystem I	*psaB, psaA, psaI, psaJ, psaC*
Photosystem II	*psbA, psbB, psbD, psbE, psbF, psbH, psbI, psbJ, psbK, psbL, psbM, psbN, psbT, psbZ, psbC*
Cytochrome b/f complex	petA, petB*, petD*, petG, petL, petN
Subunits of ATP synthase Protease	atpA, atpB, atpE, atpF*, atpH, atpI clpP**
The large subunit of rubisco	*rbcL*
NADH dehydrogenase	ndhA*, ndhB*(2), *ndhC, ndhD, ndhE, ndhF, ndhG, ndhH, ndhI, ndhJ, ndhK*
Maturase	*matK*
Envelope membrane protein	*cemA*
Acetyl-CoA carboxylase	*accD*
Synthesis gene	*ccsA*
Open reading frames (ORF, *ycf*)	*ycf*1, *ycf*2(2), *ycf*3**, *ycf*4, *ycf*15(2),

* Indicates gene with one intron and ** indicates gene with two introns. (×2) indicates that the number of the repeat unit is two. The *rps12* gene is trans-spliced.

**Table 2:** General features of the *Toddalia* chloroplast genomes compared in this study.

Features	*T. asiatica* 002151	*T. asiatica* 003103	*T. asiatica*
Total cp genome size (bp)	158,508	158,508	158,434
Length of LSC (bp)	86,162	86,162	86,132
Length of IR (bp)	27,007	27,007	27,008
Length of SSC (bp)	18,332	18,332	18,286
Total GC content (%)	38.5	38.5	38.5
GC content of LSC (%)	36.8	36.8	36.8
GC content of IR (%)	42.9	42.9	42.9
GC content of SSC (%)	33.4	33.4	33.4
Total number of genes	113	113	115
Protein encoding genes	79	79	81
tRNA genes	30	30	30
rRNA genes	4	4	4

**Table 3:** Relative variable importance percentage.

Code	Environmental Variables	Based on the AUC Metric
bio2	mean diurnal range	3.7%
bio3	Isothermality (bio2/bio7) × 100	0.9%
bio8	Mean Temperature of the Wettest Quarter	7.6%
bio9	Mean Temperature of Driest Quarter	19.9%
bio13	Precipitation of the Wettest Month	39.4%
bio14	precipitation of the driest month	2.3%
bio15	precipitation seasonality	5.3%
bio18	precipitation of warmest quarter	2%
bio19	precipitation of the coldest quarter	1.2%

## Data Availability

The assembled chloroplast genome sequences have been deposited in GenBank and accession numbers obtained (shown in Supplementary Table S1).

## Supplementary Materials

The following are available online at https://www.mdpi.com/article/10.3390/plants11020231/s1, Figure S1: Mauve alignment of the three *Toddalia* species, Figure S2: Ecological niche of *Toddalia* species, Figure S3: ROC-AUC curves of the five models. Table S1: Information of two newly sequenced plastomes, Table S2: The plastomes obtained from GenBank in this study, Table S3: The lengths of introns and exons for the splitting genes, Table S4: Relative synonymous Codon usage (RSCU) and percentage (%) RSCU analysis of *T. asiatica* 002151, *T. asiatica* 003103 and *T. asiatica*, Table S5: *Toddalia asiatica* long repeats, Table S6: SRRs present in *Toddalia* species, Table S7: Total number of SSRs repeats, Table S8: Model Mean performance (per species), using test dataset (generated using partitioning) of *Toddalia asiatica*.
