# Supplementary material for: Comparative Genomics, Phylogenetics, Biogeography, and Effects of Climate Change on Toddalia asiatica (L.) Lam. (Rutaceae) from Africa and Asia
Source: Plants (Basel). 2022 Jan 17;11(2):231. doi: 10.3390/plants11020231 (PMC8781850; doi:10.3390/plants11020231)
Supplement: Supplementary file 1 [file plants-11-00231-s001.zip › plants-1474996-SI.pdf]

Supplementary Material

Supplementary Table S1. The lengths of introns and exons for the splitting genes.

Gene	Strand	Start	End	ExonI	IntronI	ExonII	IntronII	ExonIII
trnK-UUU	-	1821	4400	38	2506	36		
rps16	-	4936	6098	42	896	225		
trnS-CGA	+	9660	10473	31	721	62		
atpF	-	12512	13869	145	797	416		
rpoC1	-	22004	24824	422	787	1612		
ycf3	-	45046	47080	129	732	228	793	153
trnL-UAA	+	49942	50574	35	546	52		
trnV-UAC	-	53628	54289	37	569	56		
clpP	-	72394	74497	71	866	291	650	226
petD	+	79013	80234	8	739	475		
rpl16	-	83637	85079	9	1032	402		
rpl2	-	86763	88280	391	657	470		
ndhB	-	97448	99661	775	681	758		
trnE-UUC	+	105211	106244	32	962	40		
trnA-UGC	+	106315	107190	37	782	57		
ndhA	+	119492	121741	553	1158	539		
trnA-UGC	-	137481	138356	37	782	57		
trnE-UUC	-	138427	139460	32	962	40		
ndhB	+	145010	147223	775	681	758		
rpl2	+	156391	157908	391	657	470		
trnK-UUU	-	1821	4400	38	2506	36		

Supplementary Table S2. Relative synonymous Codon usage (RSCU) and percentage (%) RSCU analysis of *T. asiatica 002151, T. asiatica 003103* and *T. asiatica.*

Amino Acid	Codon	Count Toddalia 002151)	RSCU	% RSCU	Count (*Toddalia 003103)*	RSC U	% RSCU	Count (*T. asiatica*)	RSCU	% RSCU	% RSCU
Phenylalanine(F)	UUU	2237	1.2		2239	1.2		2229	1.19	7.088	
	UUC	1485	0.8	7.044	1484	0.8	7.046	1516	0.81		7.329
Leucine(L)	UUA	1026	1.14		994	1.11		1029	1.17		
	UUG	1132	1.26		1124	1.25		1127	1.28		
	CUU	1196	1.33		1203	1.34		1136	1.29		
	CUC	700	0.78	10.24	698	0.78	10.20	675	0.77	10.01	
	CUA	839	0.93		838	0.93		831	0.94		9.998
	CUG	515	0.57		533	0.59		492	0.56		
Isoleucine(I)	AUU	1841	1.27		1808	1.24		1766	1.24		8.111
	AUC	1111	0.77	8.234	1096	0.75	8.261	1074	0.75	8.104	
	AUA	1399	0.96		1461	1		1442	1.01		
Methionine(M)	AUG	901	1		887	1		859	1	1.626	
Valine(V)	GUU	808	1.37	1.705	811	1.38	1.679	781	1.37	4.323	1.659
	GUC	461	0.78	4.469	470	0.8		435	0.76		
	GUA	673	1.14		659	1.12	4.444	633	1.11		4.445
	GUG	419	0.71		408	0.7		435	0.76		
Serine(S)	UCU	1077	1.39		1081	1.39		1126	1.38		
	UCC	855	1.11		883	1.14		898	1.1		
	UCA	886	1.15		864	1.11		974	1.2	9.234	
	UCG	652	0.84	8.7819	641	0.83	8.812	662	0.81		
	AGU	674	0.87		679	0.88		687	0.84		9.413
	AGC	496	0.64		508	0.65		532	0.65		
Proline(P)	CCU	730	1.12		702	1.07		648	1		4.792
	CCC	665	1.02		678	1.04		679	1.05	4.9152	
	CCA	745	1.14	4.938	775	1.18	4.957	811	1.25		
	CCG	469	0.72		464	0.71		459	0.71		
Threonine(T)	ACU	635	1.17		635	1.16		658	1.15		
	ACC	563	1.04		585	1.07		589	1.03	4.321	4.416
	ACA	627	1.15	4.114	624	1.14	4.139	651	1.14		
	ACG	349	0.64		343	0.63		385	0.67		
Alanine(A)	GCU	482	1.18		485	1.19		455	1.15		
	GCC	381	0.93	3.093	378	0.93	3.0992	408	1.03		

	GCA	470	1.15		476	1.17		448	1.13	2.999	3.092
	GCG	301	0.74		295	0.72		274	0.69		
Tyrosine(Y)	UAU	1411	1.33	4.001	1412	1.33	3.898	1399	1.33	3.975	
	UAC	703	0.67		705	0.67		701	0.67		3.966
Histidine(H)	CAU	921	1.37	2.536	991	1.41	2.661	912	1.37	2.511	
	CAC	419	0.63		415	0.59		415	0.63		2.425
Glutamine(Q)	CAA	1159	1.4	3.132	1181	1.41	3.174	1097	1.36	3.057	2.848
	CAG	496	0.6		496	0.59		518	0.64		
Asparagine(N)	AAU	1709	1.36	4.758	1732	1.36	4.813	1704	1.37	4.720	
	AAC	805	0.64		811	0.64		790	0.63		4.966
Lysine(K)	AAA	2098	1.34	5.947	2099	1.33	5.966	2077	1.33	5.891	
	AAG	1044	0.66		1053	0.67		1036	0.67		5.947
Aspartic acid(D)	GAU	1066	1.37	2.947	1067	1.37	2.946	1056	1.43	2.795	
	GAC	491	0.63		490	0.63		421	0.57		2.973
Glutamic acid (E)	GAA	1312	1.34	3.711	1303	1.33	3.713	1391	1.39		
	GAG	649	0.66		659	0.67		605	0.61	3.777	3.909
Cysteine (C)	UGU	691	1.27	2.084	672	1.26	2.019	648	1.12	2.194	
	UGC	401	0.73		395	0.74		511	0.88		2.106
Tryptophan (W)	UGG	732	1	1.385	694	1	1.313	714	1	1.351	1.336
Arginine (R)	CGU	408	0.68		414	0.7		431	0.73		
	CGC	296	0.49		298	0.5		308	0.53		
	CGA	700	1.17	6.800	694	1.17	6.726	692	1.18	6.660	
	CGG	474	0.79		472	0.8		444	0.76		
	AGA	1072	1.79		1038	1.75		1020	1.74		6.446
	AGG	643	1.07		638	1.08		624	1.06		
Glysine(G)	GGU	576	0.96		574	0.96		561	0.93		3.616
	GGC	382	0.64	3.323	404	0.68	4.525	433	0.72	4.559	
	GGA	798	1.33		768	1.28		799	1.33		
	GGG	645	1.07		645	1.08		616	1.02		
Stop codon(*)	UAA	1121	1.15		1134	1.17		1209	1.18	5.837	
	UAG	824	0.84	5.555	808	0.83	5.501	853	0.83		5.229
	UGA	990	1.01		965	1		1022	0.99		

Supplementary Table S3*:* *Toddalia asiatica* long repeats.

Repeat size (bp)	1st start	Repeat type	2nd start	location 1	location 2	Region
73	40896	F	43120	psaB	*PsaA*	LSC
55	40914	F	43138	psaB	*PsaA*	LSC
48	30944	P	30944	*IGS(petN-psbM)*	IGS(*petN-psbM*)	LSC
48	76289	P	76289	psbB	*psbB*	LSC
41	67276	F	71103	*IGS(psbE-petL)*	*rpl*20	LSC
40	110771	P	110771	*IGS(rrn5-trnR-ACG)*	*IGS(rrn5-trnR-CG)*	IRb
40	110771	F	132972	*IGS(rrn5-trnR-ACG)*	IGS(*trnN-GUU- trnR-ACG*)	IRa/IRb
40	132972	P	132972	*IGS(trnN-GUU-trnR-ACG)*	IGS(trnN-GUU- trnR-ACG)	IRb
41	100897	F	123067	*rps12*	*ndhA*	IRb/IRa
41	123067	P	142845	*ndhA*	*IGS(rps12-trnv-GAC)*	IRa
40	93484	F	93502	*Ycf2*	*Ycf2*	IRa
40	93484	P	150241	Ycf2	*Ycf2*	IRa / IRb
40	93502	P	150259	*Ycf2*	Ycf2	IRa/ IRb
40	150241	F	150259	*Ycf2*	*Ycf2*	IRb/IRa
30	69885	P	69885	*IGS(psaJ-rp133)*	*IGS(psaJ-rp133)*	LSC
30	79141	P	79141	*petD*	*petD*	LSC
34	109986	F	110018	*rrn23*	*rrn23*	IRb
34	109986	P	133731	*rrn23*	*Ycf2*	IRb/IRa
34	110018	P	133763	*rrn23*	*Ycf2*	IRb/IRa
34	133731	F	133763	*IGS(rrn5-trnR-ACG)*	IGS(*rrn5-trnR-ACG)*	IRb
30	8134	P	47262	*IGS(psbk-psbI)*	*Ycf3*	LSC
31	10321	F	10348	*IGS(trnS-GCU-trnG-UCC)*	*trnG-UCC*	LSC
33	29896	F	29928	IGS (trnC-GCU-PetN)	IGS(*trnC-GCU- PetN*)	LSC
32	8132	F	37441	IGS(psbk-psbI)	p*sbC*	LSC
32	101300	C	142455	*rps*12	*IGS (trnV-GAC- rps12)*	LSC/IRA
73	40896	F	43120	psaB	*psaA*	LSC

55	40914	P	43138	psaB	*psaA*	LSC
48	30944	F	30944	*IGS(petN-psbM)*	*IGS(petN-psbM)*	LSC
48	76289	F	76289	*psaB*	*psaB*	LSC
41	67276	F	71103	*IGS(psbE-petL)*	*rpl20*	LSC
40	110771	F	110771	*IGS(rrn5-trnR-ACG)*	*IGS(rrn5-trnR-CG)*	IRb
40	110771	P	132972	*IGS(rrn5-trnR-ACG)*	*IGS(trnN-GUU- trnR-ACG)*	IRb/IRa
40	132972	P	132972	*IGS(trnN-GUU-trnR-ACG)*	*IGS(trnN-GUU- trnR-ACG)*	IRb
41	100897	F	123067	*rps12*	*ndhA*	IRa

Supplementary Table S4: SRRs present in *Toddalia* species.

SSR number	SSR type	SSR	Size	Start	End	location	region
1	p1	(T)12	12	1736	1747	*IGS (psbA-trnK-UUU)*	LSC
2	p1	(T)11	11	2375	2385	trnK-UUU	LSC
3	p1	(A)12	12	4649	4660	trnK-UUU	LSC
4	p1	(A)11	11	6264	6274	rpl16	LSC
5	p1	(A)10	10	6767	6776	IGS (*rpl16-trnQ-UUG*)	LSC
6	p1	(T)10	10	7320	7329	IGS (rpl16-trnQ-UUG)	LSC
7	p1	(A)11	11	7746	7756	IGS (rpl16-trnQ-UUG)	LSC
8	p1	(T)10	10	8294	8303	IGS (psbK-psbI)	LSC
9	p1	(A)10	10	8447	8456	IGS (psbK-psbI))	LSC
10	p1	(A)11	11	8680	8690	*IGS (trnS-GCU- trnS-GCA)*	LSC
11	p1	(T)13	13	9282	9294	IGS (trnS-GCU- trnS-GCA)	LSC
12	p1	(A)13	13	13375	13387	atpf	LSC
13	p1	(G)10	10	15538	15547	*IGS (atpI-atpH)*	LSC
14	p1	(A)10	10	17453	17462	IGS (*rps2-rpoc2*)	LSC
15	p1	(T)11	11	19569	19579	rpoc2	LSC
16	p2	(AT)5	10	21064	21073	rpoc2	LSC
17	p1	(A)10	10	23741	23750	rpoc1	LSC
18	p1	(T)10	10	24123	24132	rpoc1	LSC
19	p1	(T)10	10	27417	27426	*rpoB*	LSC
20	c	(A)11	106	29083	29188	IGS (*rpoB-trnC-GCA)*	LSC
21	p1	(A)12	12	29824	29835	IGS (trnC-GCA-petN)	LSC
22	p1	(T)10	10	31942	31951	IGS (*psbM-trnD-GUC*)	LSC
23	p1	(T)14	14	32120	32133	IGS (*psbM-trnD-GUC*)	LSC

24	p1	(A)14	14	32554	32567	IGS (*trnD-GUC- trnY-GUA*)	LSC
25	p4	(AAAT)3	12	32704	32715	IGS (*trnD-GUC- trnY-GUA*)	LSC
26	p1	(T)11	11	33478	33488	IGS (*trnE-UUC- trnT-GGU*)	LSC
27	p1	(T)10	10	35012	35021	IGS (*trnT-GGU-psbD*)	LSC
28	p1	(G)11	11	36743	36753	*psbC*	LSC
29	c	(AT)5	114	38727	38840	IGS *(PsbZ-trnG-GCC)*	LSC
30	p4	(TAAA)4	16	45018	45033	IGS (*PsaA-Ycf3*)	LSC
31	p1	(T)10	10	45894	45903	*Ycf*3	LSC
32	p1	(A)11	11	47183	47193	IGS (*Ycf3-trnS-GGA*)	LSC
33	p1	(T)10	10	48486	48495	IGS (*rps*4*-trnt-GGU*)	LSC
34	p4	(TAAT)3	12	49504	49515	IGS (*trnt-GGU-trnL-UAA*)	LSC
35	c	(T)12	70	51107	51176	IGS (*trnF-GAA-ndhJ*)	LSC
36	p1	(T)10	10	52788	52797	*IGS (ndhc- trnV-UAC)*	LSC
37	p3	(TTA)4	12	53602	53613	IGS (*ndhc- trnV-UAC*)	LSC
38	p1	(T)11	11	56703	56713	IGS (*atpB- rbcL*)	LSC
39	p2	(TA)5	10	57075	57084	IGS (*atpB- rbcL*)	LSC
40	c	(T)11	115	60950	61064	IGS *(accD- psaI*)	LSC
41	p3	(TAA)4	12	61355	61366	IGS (*accD- psaI*)	LSC
42	p1	(T)10	10	61767	61776	IGS (*PsbI- Ycf4*)	LSC
43	p1	(T)11	11	62797	62807	IGS (*ycf4- cemA*)	LSC
44	p1	(T)11	11	67434	67444	IGS (*psbE- petL*)	LSC
45	p1	(A)10	10	68987	68996	IGS (*trnW-CAA-trnP-UUG*)	LSC
46	c	(A)11	81	69239	69319	IGS (*trnP-UUG-psaJ*)	LSC
47	p4	(TAGA)3	12	69712	69723	IGS (trnW-CAA-trnP-UUG)	LSC
48	p4	(AAAT)3	12	70399	70410	IGS (*rpl*33-*rps*18)	LSC
49	p1	(A)10	10	70897	70906	IGS (*rps18-rpl20*)	LSC
50	p3	(TTC)4	12	72067	72078	IGS (*rpl*20-*rps*12)	LSC
51	p1	(T)10	10	72978	72987	*clpP*	LSC
52	p1	(A)10	10	73143	73152	*clpP*	LSC
53	p1	(T)12	12	76562	76573	*psb*B	LSC
54	p1	(T)12	12	84295	84306	*rpl2*	LSC
55	p1	(T)14	14	85939	85952	*rpl23*	LSC
56	p3	(AAG)4	12	96318	96329	IGS (*Ycf2-trnL-CAA*)	IRa
57	p1	(T)14	14	101749	101762	IGS (*rps7-rps12*)	IRa
58	p1	(A)10	10	105584	105593	*trnE-UUC*	IRa
59	p1	(A)14	14	110785	110798	IGS (*rrn5-trnR-ACG*)	IRa
60	p1	(A)10	10	111225	111234	IGS (*trnN-ACG-trnN-GUU-Y*)	IRa
61	p1	(A)12	12	113027	113038	*Ycf*1	IRa
62	p1	(A)12	12	115536	115547	*Ycf*1	SSC
63	p4	(TATT)3	12	116258	116269	*Ycf1*	SSC

64	c	(A)11	29	116713	116741	*Ycf1*	SSC
65	p1	(T)12	12	118627	118638	IGS (*rps15-ndH*)	SSC
66	p2	(AT)5	10	120068	120077	*ndhA*	SSC
67	p1	(A)10	10	120297	120306	*ndhA*	SSC
68	c	(TA)5	28	122142	122169	*ndhA*	SSC
69	p1	(A)10	10	123680	123689	*ndhE*	SSC
70	p1	(T)11	11	129789	129799	*ndhF*	SSC
71	p1	(T)12	12	131631	131642	*Ycf1*	SSC
72	p1	(T)10	10	133435	133444	IGS (trnN-GUU-trnR-ACG)	IRb
73	p1	(T)14	14	133871	133884	IGS (*trnR-ACG-rrn5*)	IRb
74	p1	(T)10	10	139076	139085	*trnE-UUC*	IRb
75	p1	(A)14	14	142907	142920	IGS (*Ycf*15-*rp`s*12)	IRb
76	p3	(CTT)4	12	148340	148351	IGS (trnL-CAA- *ycf2)*	IRb

Supplementary Table S5: Total number of SSRs repeats.

SSRs	*T. asiatica 002151*	*T. asiatica 003103*
A/T	60	60
C/G	2	2
AT/AT	6	6
AAG/CTT	3	3
AAT/ATT	4	4
AAAT/ATTT	4	4
AACT/AGTT	1	1
AATT/AATT	1	1
ACAT/ATGT	1	1
AGAT/ATCT	1	1
Total no. of SSRs	83	83

Supplementary Table S6: Model Mean performance (per species), using test dataset (generated using partitioning) of *Toddalia asiatica*

Methods	AUC	COR	TSS	Deviance
Maxent	0.98	0.88	0.86	0.61

Supplementary Table S7. Information of two newly sequenced plastomes.

Species	Sample locality	Voucher (Herbarium)	GenBank accession
Toddalia asiatica 002151	Mt. Kenya, Kenya	SAJIT-002151	OK127881
Toddalia asiatica 003103	Mt. Kenya, Kenya	SAJIT-003103	OK127880

Supplementary Table S8. The plastomes obtained from GenBank in this study.

Species	GenBank accession
Phellodendron chinense	NC050949
Tetradium daniellii	MW542638
Toddalia asiatica	MW542637
Ailanthus altissima	MG799542
Atalantia kwangtungensis	MH329190
Casimiroa edulis	MN539263
Citrus aurantiifolia	KJ865401
Citrus hongheensis	MT880607
Clausena excavate	KU949003
Glycosmis mauritiana	KU949004
Glycosmis pentaphylla	KU949005
Leitneria floridana	KT692940
Melicope pteleifolia	MW046256
Merrillia caloxylon	KU949006
Micromelum minutum	KU949007
Murraya koenigii	KU949002
Phellodendron amurense	NC_035551
Ruta graveolens	MN326012
Tetradium ruticarpum	MT134114
Zanthoxylum acanthopodium	MT795653
Zanthoxylum armatum	MT990984
Zanthoxylum bungeanum	KX497031
Zanthoxylum calcicola	MT990983
Zanthoxylum madagascariense	MN968551
Zanthoxylum nitidum	MK613864
Zanthoxylum oxyphyllum	MT990980
Zanthoxylum paniculatum	MN968552
Zanthoxylum piasezkii	MT990979
Zanthoxylum pinnatum	MN968553

Zanthoxylum piperitum	KT153018
Zanthoxylum schinifolium	KT321318
Zanthoxylum simulans	MF716524
Zanthoxylum tragodes	MN968554

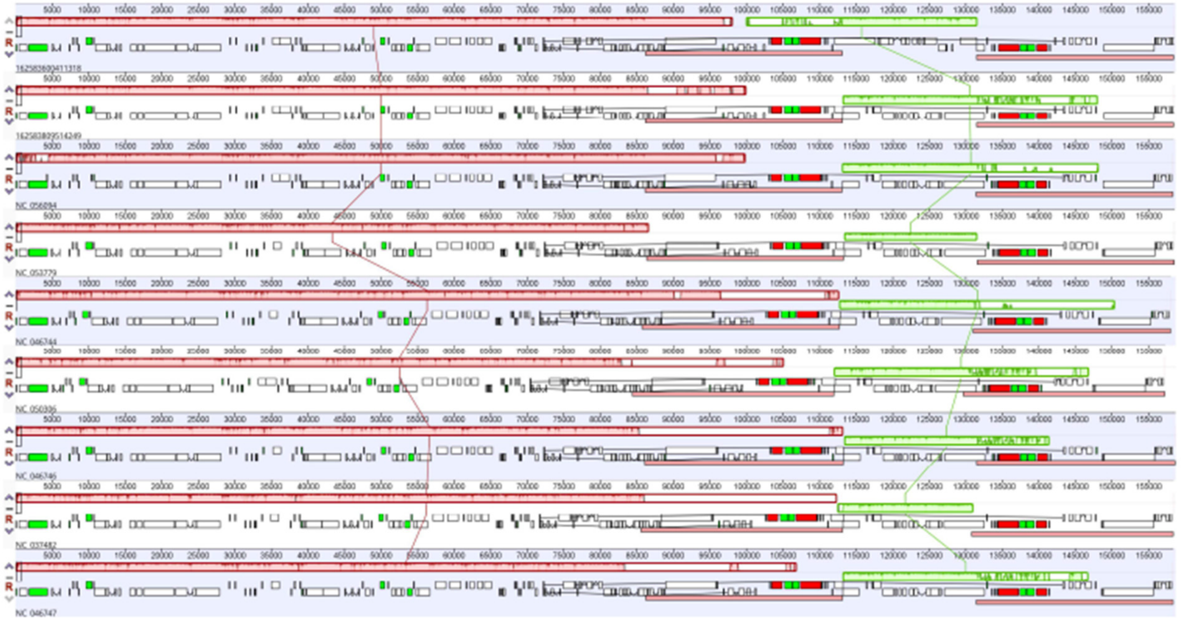

Supplementary Figure S1: Mauve alignment of the three *Toddalia* species.

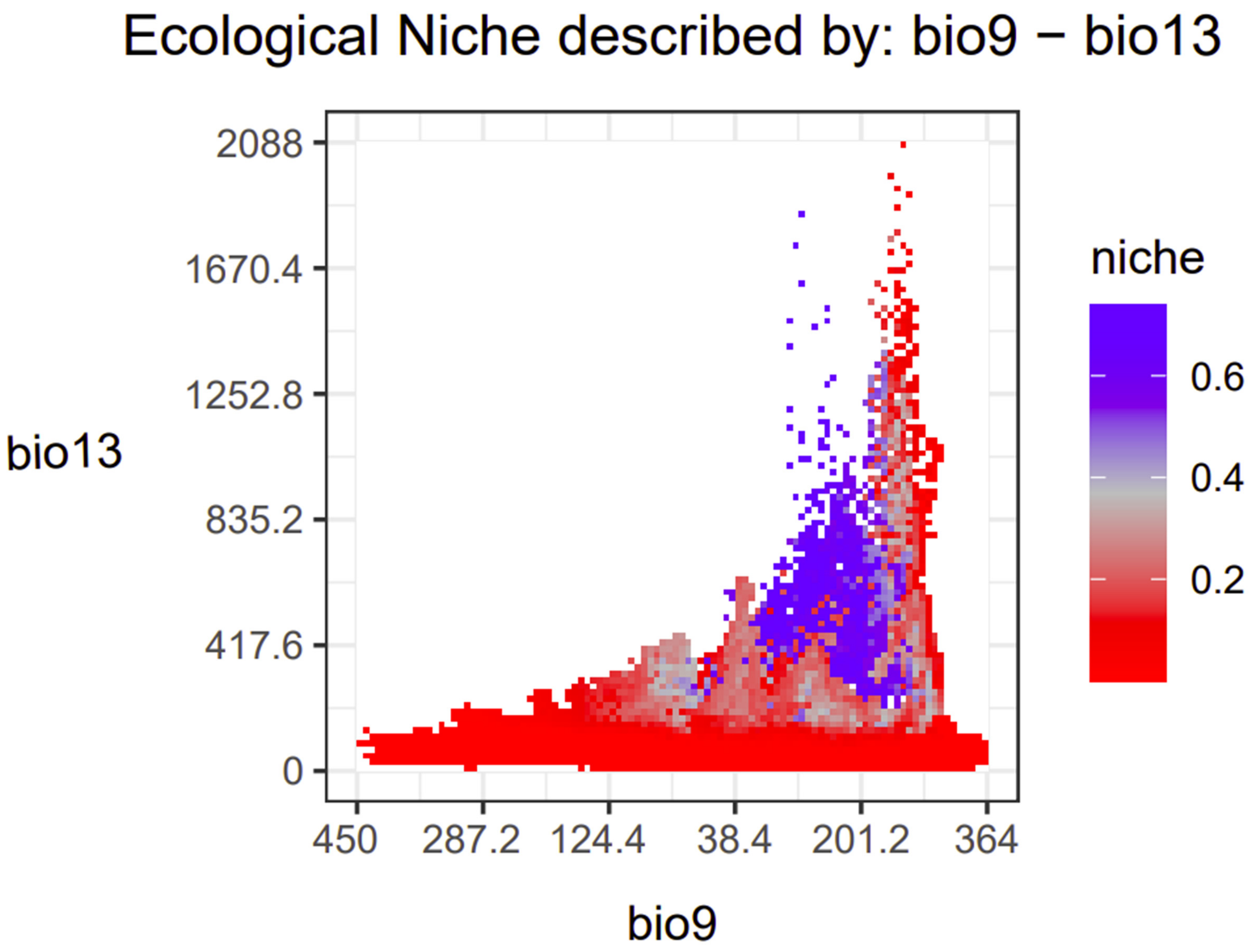

Supplementary Figure S2: Ecological niche of *Toddalia* species.

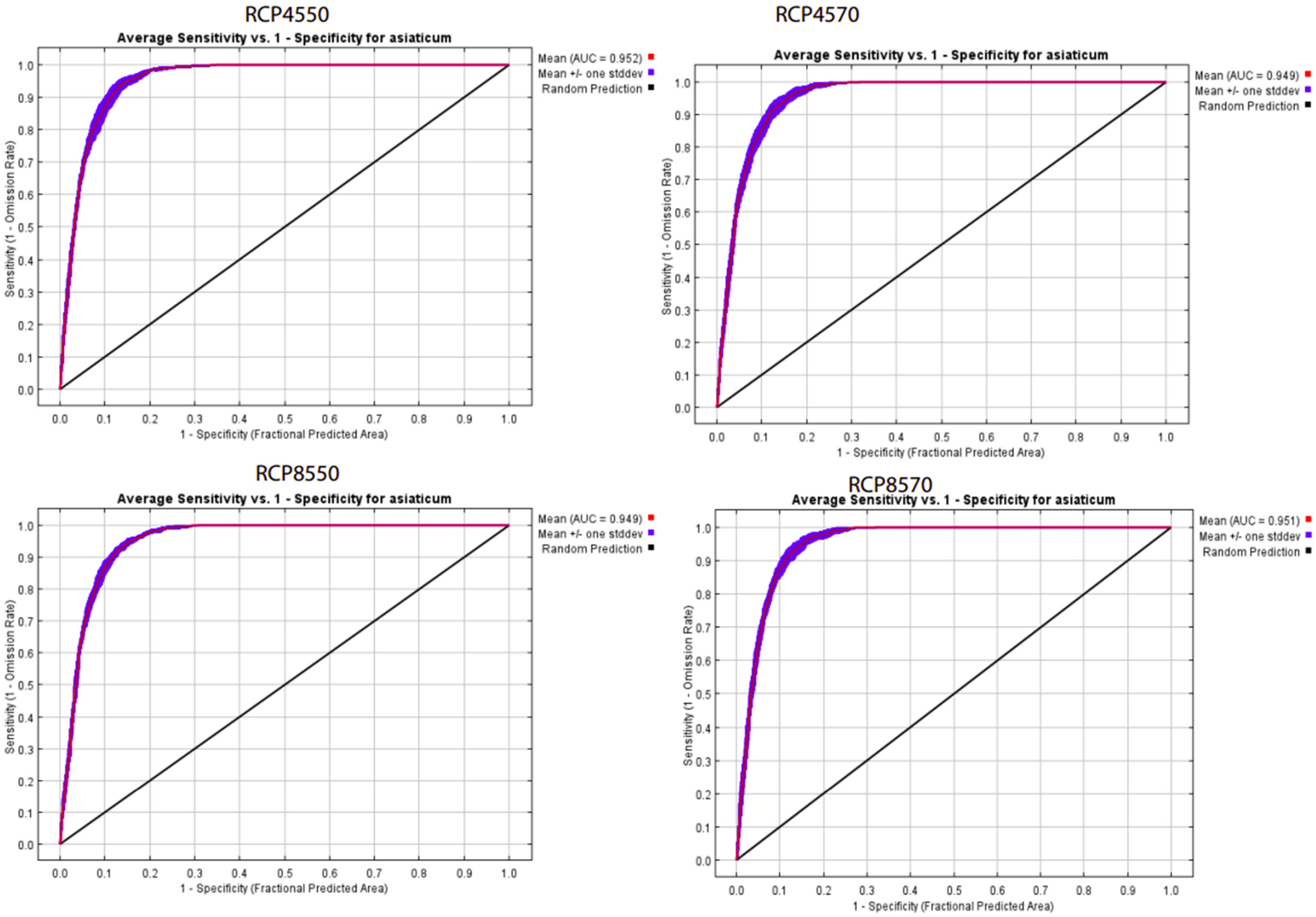

Supplementary Figure S3: ROC-AUC curves of the five models.
